# Evaluation of the suitability of Gardenia blue pigment derived from *Gardenia jasminoides* Ellis (Rubiaceae) as a dental plaque disclosant

**DOI:** 10.1002/cre2.634

**Published:** 2022-07-13

**Authors:** Im‐hee Jung, Young Sun Hwang

**Affiliations:** ^1^ Department of Dental Hygiene, College of Health Science Eulji University Seongnam Korea

**Keywords:** dental plaque, oral hygiene, periodontitis, pigmentation

## Abstract

**Objective:**

Dental disclosants are used to distinguish the amount and location of dental plaque. Therefore, dental disclosants are useful for dental plaque management and effective in motivating oral care. After reports on the cytotoxicity and carcinogenesis of dental disclosants containing erythrosine, many natural pigments for dental disclosants have been suggested. However, there are insufficient ingredients with proven biocompatibility for human subjects. The purpose of this study was to explore the suitability of Gardenia blue pigment as a dental disclosant.

**Materials and Methods:**

Natural Gardenia blue pigment was used as the dental disclosant experimental group and 2Tone was used as the control group. The homogeneity of the panelists in the groups was identified by measuring the gingivitis index and dental plaque index of the subjects before the experiments. The degree of pigmentation on the tooth surface was observed immediately after coloring and after 1 h. The remaining pigment on the dental surface was also monitored after brushing the teeth. In the panelist test, the taste and sensation of the pigment were examined, and the overall preference for the pigment as a dental disclosant was examined.

**Results:**

After coloration of the tooth surface, neither the natural Gardenia blue pigment nor 2Tone imparted any special taste or sensation. The coloration of dental plaque with Gardenia blue pigment was similar to that of 2Tone, and the difference in the degree of coloration between Gardenia blue pigment and 2Tone was not statistically significant. The residual degree of pigmentation after 1 h of coloring was similar in both groups, but most of it was removed by brushing. There was no statistically significant difference in the overall preference of Gardenia blue pigment over 2Tone.

**Conclusions:**

The results of this study prove that natural Gardenia blue pigment could be a suitable dental disclosant in terms of pigmentation and preference.

## 1 INTRODUCTION

Oral microorganisms are a major risk factor for dental caries and periodontal disease. Dental plaque, a colorless or whitish substance that builds up around the teeth and gums, is made up of billions of bacteria and is strongly associated with oral disease. Therefore, dental plaque control is very important in preventing gum diseases caused by oral microorganisms. Dental disclosants can stain cellular plaque containing oral microorganisms and is very useful in distinguishing the location and degree of dental plaque. Education on tooth brushing using dental disclosants is very effective and provides strong motivation for oral care (Lee & Kang, 2018).

However, a commonly used dental disclosant in dental clinics is the tar pigment erythrosine, Red No. 3, which has been strongly associated with cytotoxicity and carcinogenesis in in vivo animal experiments, and the Food and Drug Administration (FDA) recommends prohibiting its use (Carlson, 1977; Jennings et al., 1990; Watanabe et al., 1986; Yankell & Loux, 1977). Therefore, various alternative ingredients for erythrosine, including those derived from natural sources, have been suggested, but have not yet been applied in practice (Adnan et al., 2019; Amaliya et al., 2019; Febriyanti et al., 2018). In addition, only the degree of dental surface pigmentation by the ingredients was mainly reported, while biocompatibility studies in humans are insufficient. To urgently identify alternative dental disclosants, a biocompatibility study of ingredients with effective dental plaque pigmentation is required.

In a previous study, we verified the pigmentation superiority of natural Gardenia blue pigment derived from *Gardenia jasminoides* Ellis (Rubiaceae) in bovine teeth and tongues (Kim et al., 2021). Since Gardenia blue pigment had low cytotoxicity, it was hypothesized that if its pigmentation degree was approximately 2Tone, it could be an effective substitute for disclosant. Therefore, in this study, the degree of dental plaque pigmentation and preference of Gardenia blue pigment derived from *Gardenia jasminoides* Ellis (Rubiaceae) was investigated in the human oral environment. The result suggests the suitability of natural product‐derived ingredients as dental disclosants.

## 2 MATERIALS AND METHODS

### 2.1 Pigment materials

The natural Gardenia blue pigment extracted with water from *Gardenia jasminoides* Ellis (Rubiaceae) was purchased from Nafarm Co. Ltd. (Seongnam, Korea). 2Tone (F.D.&C. Green No.3, F.D.&C. Red No.3; Young Dental Manufacturing, Earth City, MO, USA) was used as controls (Block et al., 1972). The product stock solution was used in the experiment.

### 2.2 Participant selection

Twenty adults, regardless of gender, participated in the panel. The exclusion criteria included bleeding in the gums or oral cavity, and less than a month after scaling before the experiment. They were randomized into two groups of 10 people each. Starting 3 h before the experiment, the panelists in the study did not brush their teeth or perform oral hygiene. The sample size was calculated using G*Power 3.1. sampling software (Heinrich‐Heine‐University Dusseldorf, Dusseldorf, Germany) with an alpha error probability of 5% and power of 80% for the two independent study groups. The human experiment was approved by the Institutional Review Board (IRB) of Eulji University (approval no. EU20‐12).

### 2.3 Evaluation of gingivitis and dental plaque indexes in the participants

Gingivitis was assessed by the Löe−Silness gingival index in the buccal sites of the upper/lower and left/right first molars, maxillary right central incisors, and mandibular left central incisors and scored according to the inflammation degree as no inflammation (0); mild, mild gum color change and swelling (1); moderate, pronounced redness and swelling (2); and severe, ulceration and bleeding (3) (Löe & Silness, 1963). The dental plaque index was measured using the O'Leary index according to the degree of adhesion of the bacterial film as no dental plaque (0); dotted to the gingival margin (1); linear to the gingival margin at ≦1 mm (2); adhered to the cervical 1/3 (3); adhere to the cervical 2/3 (4); attached more than 2/3 of the cervical (5) (O'Leary et al., 1972). All oral examinations and assessments were evaluated by the dentist (J. S. Kim, Seoul Hana Dental Clinic, Seongnam, Korea).

### 2.4 Evaluation of pigmentation on human mandibular incisors

The evaluation of dental plaque staining was conducted at the lingual site of the four mandibular incisors. The staining strength of dental plaque was evaluated as very dark (4), deep (3), light (2), and very light (1). The grades for the four mandibular incisors were summed as the dental plaque index of each subject. The pigmentation remaining after 1 h of application and brushing was also evaluated. The area was photographed, and the relative coloration was measured in equal pixel areas using ImageJ (NIH). The institutional review board (IRB) approved this test (EU20‐12).

### 2.5 Taste, sensation, and preference tests

Taste, sensation, and preference for the pigment were tested during the pigmentation process. The degree of contrast between the gum‐tooth and the pigment and overall preference for the pigment were evaluated as good (4), moderate (3), bad (2), and very bad (1).

### 2.6 Statistics

Statistical analyses were conducted using InStat GraphPad Prism ver. 5.01 statistical software (GraphPad Software, Inc., San Diego, CA, USA). The results are expressed as the means ± SD. Nonparametric Kruskal−Wallis test with Dunn's post hoc analysis was employed for multiple comparisons. Nonparametric Wilcoxon's signed‐rank test was used for coloring tests of natural pigments on mandibular incisors. *p* < .05 was considered to indicate a statistically significant difference.

## 3 RESULTS

### 3.1 Oral assessment of panelists

The age, gender, gingivitis index, and dental plaque index of the panelists who participated in the suitability evaluation are indicated in Table 1. Ten panelists participated in each group with similar gender ratios. The gingivitis index was 0.5 ± 0.52 for the Gardenia blue pigment group and 0.3 ± 0.48 for the 2Tone group. There were no statistically significant differences in the gingivitis index between the two groups (*p* = .388). The dental plaque index was 0.7 ± 0.67 for the Gardenia blue pigment group and 1.0 ± 0.81 for the 2Tone group, also without significant differences between the two groups (*p* = .382). These results suggest that the dental environment of the panelists in the two groups was similar and suitable for a study comparing the biocompatibility of dental disclosants.

**Table 1:** General characteristics of the participants

Characteristic	Group (%)		*p* value
	2Tone (*n* = 10)	Gardenia blue pigment (*n* = 10)	
Age (years)			
20−39	7 (70)	8 (80)	
40−59	2 (20)	2 (20)	
60−70	1 (10)	0 (0)	
Gender			
Male	5 (50)	4 (40)	
Female	5 (50)	6 (60)	
Gingivitis index	0.3 ± 0.48	0.5 ± 0.52	.388
Dental plaque index	1.0 ± 0.81	0.7 ± 0.67	.382

### 3.2 Pigmentation evaluation of mandibular incisors

**Figure 1:**
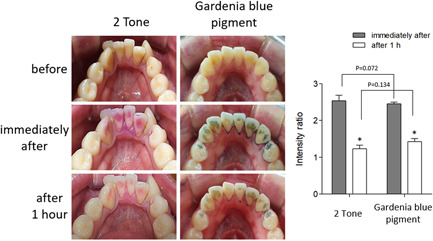
Pigmentation evaluation on mandibular incisors. Gardenia blue pigment or 2Tone was applied evenly to the lingual side of the four mandibular incisors and the mouth was washed with water three times after 10 s. Images were captured with a digital camera and the relative dye intensity before and after the experiment was measured in equal pixel area using the ImageJ program and plotted as a graph. *p < .01 versus immediately after.

The Gardenia blue pigment or 2Tone moistened with a cotton swab was applied evenly to the lingual side of the four mandibular incisors, the mouth was washed with water three times after 10 s, and pigmentation was observed. As shown in Figure 1, compared to before the application, the Gardenia blue pigment showed dark blue pigmentation, distinguishing it from the teeth and gums, and it effectively pigmented not only the gingival margin and cervical region but also dental plaque on the tooth surface. 2Tone pigmented in red and blue also effectively stained dental plaque on the tooth surface as well as the gingival margin and cervical region. As a result of comparing the degree of pigmentation using ImageJ, the Gardenia blue pigment group was 2.5 ± 0.47 and the 2Tone group was 2.7 ± 1.05. There was no statistically significant difference between the two groups (*p* = .072).

One hour after application, the degree of pigmentation in the Gardenia blue pigment group was 1.42 ± 1.03 and it was 1.37 ± 0.63 in the 2Tone group, without a statistically significant difference between the two groups (*p* = .134). Both pigments remained pigmented 1 h of application. However, no residual pigmentation was observed after brushing the teeth in both groups (data not shown).

### 3.3 Taste, sense, and preference tests

The taste, sensation, and preference for Gardenia blue pigment or 2Tone were evaluated (Table 2). The panelists in both groups did experience any special or unpleasant taste. Also, aching or tingling sensations were not significantly detected. The contrast preference between the gum‐tooth and the pigment was 3.6 ± 0.101 in the Gardenia blue pigment group and 3.9 ± 0.488 in the 2Tone group, and there was no statistically significant difference between the two groups (*p* = .232) (Table 3). Based on this, the overall preference for the pigment was 3.9 ± 0.322 for the Gardenia blue pigment group and 3.89 ± 0.177 for the 2Tone group, and there was no statistically significant difference between the two groups (*p* = .660). These results suggest that Gardenia blue pigment is an effective ingredient as a dental disclosant.

**Table 2:** Taste and sensation test

Characteristic	Group (%)	
	2Tone (*n* = 10)	Gardenia blue pigment (*n* = 10)
Taste		
Unpleasant	0 (0)	0 (0)
Usual	1 (10)	2 (20)
Pleasant	0 (0)	1 (10)
Tasteless	9 (90)	7 (70)
Sensation		
Aching	0 (0)	0 (0)
Tingling	1 (10)	0 (0)
Non	9 (90)	10 (100)

**Table 3:** Preference test

Characteristic	Group		*p* value
	2Tone (*n* = 10)	Gardenia blue pigment (*n* = 10)	
Contrast preference (Gum‐Tooth‐Pigment)	3.9 ± 0.488	3.6 ± 0.101	.232
Overall preference	3.8 ± 0.177	3.9 ± 0.322	.660

## 4 DISCUSSION

In our previous study, we confirmed that Gardenia blue pigment had low cytotoxicity to human oral cells (Jung et al., 2020). In addition, pigmentation with Gardenia blue pigment was also effective on bovine teeth surfaces (Kim et al., 2021). However, since no biocompatibility study has been performed in humans, this study investigated the suitability of Gardenia blue pigment as a natural ingredient for dental disclosant in the human oral environment.

An effective dental disclosant should selectively stain the dental plaque without unnecessary staining. The two‐tone disclosing solution, which is widely used in dental clinics, stains not only the dental plaque but also the entire oral cavity, especially the gums and tongues. In addition, the two‐tone disclosing solution contains Red No.3 erythrosine, which has been reported to cause thyroid cancer in mice experiments and has strong cytotoxicity (Carlson, 1977; Jennings et al., 1990; Watanabe et al., 1986; Yankell & Loux, 1977). Consequently, in 1990, FDA banned the use of erythrosine in water‐insoluble form (lake) and water‐soluble form (dye) in external drugs and cosmetics (FDA, 1990). 2Tone, a two‐tone dye mixed with erythrosine and Fast Green or Brilliant Blue, is effective in staining dental plaque and has the advantage of coloring thicker (older) dental plaques in blue and thinner (newer) dental plaque in red and it is used worldwide (including Korea and the United States). Currently, 2Tone without erythrosine is available. Nevertheless, there is a steady demand for effective alternative dental disclosant ingredients with low cytotoxicity that are effective in pigmenting dental plaque.

In this study, to determine the usefulness of Gardenia blue pigment as a dental disclosant ingredient, we compared Gardenia blue pigment with 2Tone. The effect of the disclosing solution on the tooth surface was minimized by inducing the formation of acquired pellicles by restricting toothbrushing for 3 h before the experiment. The results showed that Gardenia blue pigment stained dental plaque to a degree and duration equal to that of 2Tone. Most of the remaining pigmentation was removed by brushing the teeth after 1 h for both Gardenia blue pigment and 2Tone groups. Unlike the results of the bovine tongues and teeth pigmentation study. Most of the remaining pigmentation was removed by brushing the teeth after 1 h for both Gardenia blue pigment and 2Tone groups. Unlike the results of the bovine tongue and teeth pigmentation study (Kim et al., 2021), Gardenia blue pigment effectively and selectively stained the dental plaque and gum staining was limited. Gardenia blue pigment taste was not an unpleasant taste and did not cause a sensation that would decrease preference for the agent. In particular, the dental plaque stained by Gardenia blue pigment was easy to distinguish from the gums and teeth, so the overall preference was not different from that of 2Tone.

Dental caries and periodontal disease have a high prevalence of over 90% and have been ranked among the top most frequent outpatient diseases for the past 10 years (http://opendata.hira.or.kr/op/opc/olapHifrqSickInfo.do). They are caused by the low pH of the various proteolytic enzymes around cellular dental plaque containing oral microorganisms. Therefore, modulating the growth of oral microorganisms and maintaining a natural pH in the oral environment is helpful in the management of dental caries and periodontal disease. Dental disclosants are very useful in distinguishing the location and amount of dental plaque. Therefore, it will be effective for continuous oral health not only at the dental clinic but also by using dental disclosants when necessary.

In this study, we investigated the suitability of Gardenia blue pigment with low cytotoxicity and effective dental plaque pigmentation. The suitability of Gardenia blue pigment was found to be similar to that of 2Tone. Recruitment panelists for the suitability test were not easy due to COVID‐19, so the test evaluation was conducted with a limited number of subjects. However, the study confirmed that Gardenia blue pigment was useful as a dental disclosant. Since Gardenia blue pigment is an approved natural food coloring ingredient, it will be useful in the development of oral hygiene products including dental disclosants.

## AUTHOR CONTRIBUTIONS

*Conceptualization*: Im‐hee Jung and Young Sun Hwang. *Experiments and data acquisition*: Im‐hee Jung and Young Sun Hwang. *Formal analysis*: Im‐hee Jung and Young Sun Hwang. *Funding*: Young Sun Hwang. *Supervision*: Young Sun Hwang. *Writing*—*original draft*: Im‐hee Jung and Young Sun Hwang. *Writing*—*review and editing*: Im‐hee Jung and Young Sun Hwang.

## CONFLICT OF INTEREST

The authors declare no conflict of interest.

## Data Availability

The data supporting the findings of this study are available from the corresponding author upon reasonable request.
